# The NIST High Accuracy Scale for Absolute Spectral Response from 406 nm to 920 nm

**DOI:** 10.6028/jres.101.015

**Published:** 1996

**Authors:** T. C. Larason, S. S. Bruce, C. L. Cromer

**Affiliations:** National Institute of Standards and Technology, Gaithersburg, MD 20899-0001

**Keywords:** absolute spectral response, cryogenic radiometer, light-trapping detectors, measurements, optical power, scale, silicon photodiode, quantum efficiency, quality system

## Abstract

We describe how the National Institute of Standards and Technology obtains a scale of absolute spectral response from 406 nm to 920 nm. This scale of absolute spectral response is based solely on detector measurements traceable to the NIST High Accuracy Cryogenic Radiometer (HACR). Silicon photodiode light-trapping detectors are used to transfer optical power measurements from the HACR to a monochromator-based facility where routine measurements are performed. The transfer also involves modeling the quantum efficiency (QE) of the silicon photodiode light-trapping detectors. We describe our planned quality system for these measurements that follows ANSI/NCSL Z540-1-1994. A summary of current NIST capabilities based on these measurements is also given.

## 1. Introduction

Many radiometric, photometric, and colorimetric applications require the determination of the absolute spectral power response function of photodetectors. The absolute spectral power response is the ratio of the photodetector’s output (in amperes or volts) to the spectral radiant flux (watts) input to the photodetector. The absolute spectral power response is also referred to simply as the absolute spectral response. Accurate measurement of absolute spectral power responsivity of photodetectors has been a service provided by the Radiometric Physics Division at NIST for over 10 years. NIST has recently made changes to improve and expand the absolute spectral power response measurements provided to its customers. The most significant change is to base the measurements on the NIST High Accuracy Cryogenic Radiometer (HACR) [1].

The HACR is the U.S. primary standard for optical power. The scale of absolute spectral power response is transferred by silicon photodiode light-trapping detectors (hereafter referred to as trap detectors in this paper) [2] to working standards used with the NIST Visible to Near Infrared Spectral Comparator Facility (hereafter referred to as the comparator in this paper) where calibration service measurements are performed. This paper will briefly review the HACR, trap detectors [1, 2], and the calibration of the working standards.

The purpose of this paper is to introduce the new HACR based absolute spectral power responsivity scale in the 406 nm to 920 nm spectral range and the calibration method of photodetectors for customers. For this paper the absolute spectral power responsivity scale in the 406 nm to 920 nm spectral range will be referred to as the “visible” detector scale^1^ or just the detector scale. This paper will also present the recently expanded list of absolute spectral power responsivity measurement services provided by NIST and the enhancements to its quality system to comply with ANSI/NCSL Z540-1-1994 [3].

## 2. Background

Various techniques have been employed to determine the photodetector absolute spectral power responsivity function [4, 5]. In the late 1970s room-temperature electrical substitution radiometers (ESR), also known as electrically calibrated radiometers (ECR), were used in conjunction with lasers [6, 7] as the detector scale base. Uncertainties were reported on the order of 1.5 % to 5 % (3 standard deviation estimate^2^) [8] over the spectral range from 390 nm to 1100 nm [9]. A major advancement came in the early 1980s with the silicon photodiode self-calibration techniques and the subsequent introduction of 100 % quantum efficient (QE) photodiodes [10] and their use as the basis for detector calibrations. These photodiodes were used in the development of the United Detector Technologies (UDT) QED-200^3^ [11], which is a light-trapping device constructed of multiple windowless 100 % QE silicon photodiodes. The QED-200 had a limited spectral range (usually 400 nm to 750 nm) where it operated with 100 % QE, and suffered from limited dynamic range due to the relatively high bias currents used. The absolute spectral power response was transferred to customers at this time via the Detector Response Transfer and Intercomparison Program (DRTIP). Customers would rent a radiometer package from NIST and transfer the detector scale to their working standard(s). The scale uncertainty was typically 0.8 % to 6.0 % (3 standard deviation estimate) over the spectral range from 250 nm to 1100 nm [12].

A second generation trap detector (very similar to the commercially available Graseby Optronics QED-150) was later used as the base for the NIST detector scale [13]. Both of these trap detectors are constructed with a different type of silicon photodiode (Hamamatsu S1337-1010). They do not have 100 % QE, but the QE could be measured with the QED-200, and extrapolated with high accuracy over a large spectral range from 400 nm to 900 nm [14]. Uncertainties were reported on the order of 0.33 % to 1.0 % (3 standard deviation estimate) over the spectral range from 250 nm to 1100 nm (between 200 nm and 250 nm the uncertainty was reported as 5.0 %). The next advancement came in the late 1980’s when cryogenic ESR’s were reported with improved uncertainties over the 100 % QE detector based measurements [15,16,17].

## 3. HACR Calibration of Silicon Photodiode Trap Detectors

The HACR is the U.S. primary standard for optical power. Cryogenic radiometers are currently used as the primary standard for optical power at other national laboratories [18, 19]. The HACR will be briefly reviewed here [1]. The cryogenic radiometer was constructed to improve the accuracy and spectral range of the NIST primary standard for optical power. The HACR is an ESR that operates by comparing the temperature rise induced by optical power absorbed in a cavity to the electrical power needed to cause the same temperature rise by resistive (ohmic) heating. This links the measurement of optical power to the watt. By operating at cryogenic temperatures (≈ 5 K) instead of room temperature several advantages are gained. The heat capacity of copper is reduced by a factor of 1000 thus allowing the use of a relatively large cavity with a time constant of ≈ 4 min. Also the thermal radiation emitted by the cavity or absorbed from the surroundings is reduced (factor of ≈ 10^7^), which eliminates the radiative effects on the equilibrium temperature of the cavity. Finally, the cryogenic temperature allows the use of superconducting wires to the heater that removes the nonequivalence of optical and electrical heating because of the heat dissipated in the wires. The relative expanded uncertainty of the NIST HACR measurements is 0.021 % (coverage factor *k* = 2) [8] at ≈ 1 mW. The largest components of the uncertainty are those due to the systematic correction for the Brewster angle window transmission and the random error associated with the cavity temperature measurement.

There are a few drawbacks for making routine measurements of photodetectors with the HACR. The measurements with the HACR are very time consuming, typically taking several days for each wavelength. The measurement wavelengths are limited to the available laser wavelengths. The power levels produced for the highest accuracy measurements with the HACR can be higher than is desired for typical radiometric applications. Therefore a practical means of disseminating the detector scale is to transfer the optical power scale to another facility. Trap detectors make excellent transfer standards since they are stable and have uniform, responsivity, good linearity and low noise [2,15,19].

**Fig. 1:**
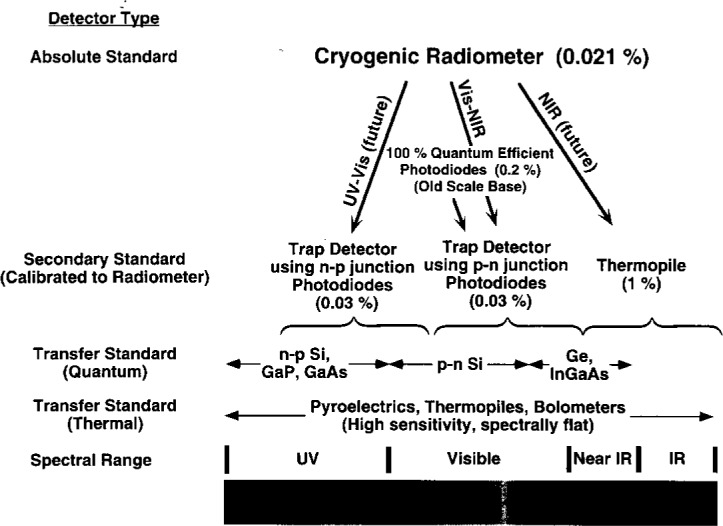
Schematic diagram of the detector scale transfer.

In transferring the detector scale, the HACR was used to determine the external quantum efficiency (EQE) of two trap detectors. These trap detectors were calibrated at nine laser wavelengths between 406 nm and 920 nm. The reflectance of each trap detector was measured in this spectral range allowing the internal quantum efficiency (IQE) to be determined. Interpolation was done using models of the IQE data, and the EQE for the entire spectral range was determined with a relative combined standard uncertainty of 0.03 % [2]. Figure 1 shows the detector scale transfer schematically.

## 4. Trap Detectors and Silicon Photodiode Working Standards

The NIST monochromator based spectral comparator facility (SCF) is used to disseminate a continuous scale of absolute spectral power response. The visible to near infrared working standards are four Hamamatsu S1337-1010BQ silicon photodiodes with fused quartz windows, which are tested for spatial responsivity uniformity and spectral response stability [19, 20]. These are the same type of photodiodes that are provided by NIST as transfer standards for the visible and near infrared spectral region. The working standards are used for all routine measurements as they are easier to align in the focal plane. Trap detectors can be used with the SCF to provide higher accuracy measurements when required.

**Fig. 2:**
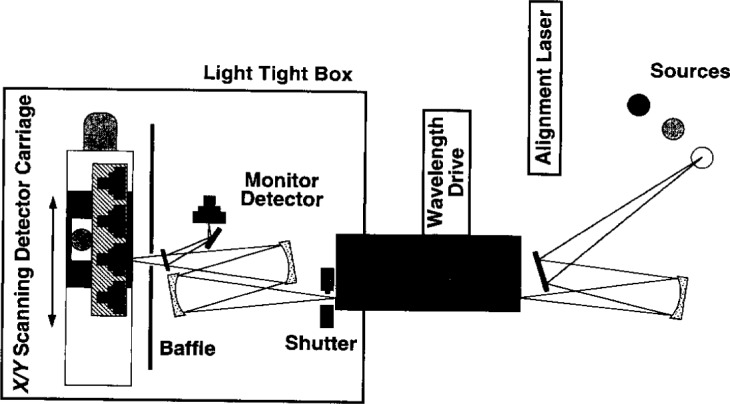
Visible to near infrared spectral comparator facility. Values in parentheses are relative expanded uncertainties (*k* = 2).

The absolute spectral power response scale is transferred from the trap detectors to working standards using the comparator. A separate ultraviolet (UV) spectral comparator system that will be described elsewhere is used with a spectrally flat pyroelectric detector to extend the scale to 200 nm. The pyroelectric detector is also used to extend the scale to 1800 nm in the infrared using germanium photodiode working standards. As shown in Fig. 2, the SCF is a monochromator based comparator system consisting of a prism-grating double monochromator using a 100 W quartz-halogen lamp as the source. The working standards and test photodetectors are positioned in the focal plane of the optical beam with *X*-*Y* translation stages. The optical beam is formed with a pair of spherical mirrors that image (with 1:1 magnification) the exit aperture of the monochromator. Mirrors are used to prevent chromatic aberrations, and the astigmatism of the spherical mirrors is corrected (to first order) by tilting the second mirror perpendicular to the plane of the first mirror [21]. A beamsplitter and monitor detector are used during the measurements and by simultaneously sampling the signals from the detector and monitor any drift in the source is removed. The signals from the working standards, trap detectors, and test photodetectors are measured unbiased (the photovoltaic or short-circuit mode) with calibrated transimpedance amplifiers [22, 23]. The entire measurement and data reduction process is computer controlled.

Each of the four working standards are measured against both trap detectors. The typical measurement is with one trap detector and two working standards placed and aligned in the comparator. Alignment is accomplished by retroreflecting a He-Ne beam back onto itself. The beam is also focused onto the working standards or the position of the last (third) detector in the trap detectors (positioning of the trap detectors is critical for this f/9 optical system to insure the beam does not overfill any of the trap detector’s photodiodes). An automated computer routine is used to determine the center of the active area for each detector, including the trap detectors. The comparison consists of scanning the monochromator through the desired spectral range at specified wavelength intervals for each detector. This process is then repeated three times for each working standard and trap detector combination. The absolute spectral power response of each working standard in the 406 nm to 920 nm spectral range is determined by comparison with the trap detectors, using the following measurement equation:

$$s(\lambda )=\frac{\frac{V_{w}(\lambda )}{V_{m_{1}}(\lambda )}}{\frac{V_{t}(\lambda )}{V_{m_{2}}(\lambda )}}\times s_{t}(\lambda )\times \frac{G_{t}}{G_{w}},$$ (1)

where *s*(λ) is the absolute spectral power response of the working standard; *V*_w_(λ) is the signal from the working standard; $V_{m_{1}}(\lambda )$ is the signal from the monitor simultaneous with *V*_w_(λ); V_t_(λ) is the signal from the trap detector; $V_{m_{2}}(\lambda )$ is the signal from the monitor simultaneous with *V*_t_(λ); *s*_t_(λ) is the absolute spectral power response determined from the interpolated EQE; *G*_t_ is the calibrated amplifier gain for the trap detector and; *G*_w_ is the calibrated amplifier gain for the working standard.

The absolute spectral power response of each working standard is the weighted mean of the measurements with each of the trap detectors. The amplifier gain for both the working standards and trap detectors is 10^6^. The optical power used for these measurements in the comparator is typically less than 1 μW.

## 5. Uncertainties

The components of uncertainty in the absolute spectral power response scale are described following the method outlined in Ref. [24]. The relative standard uncertainty components for these measurements are given in Table 1. The uncertainty due to the measurement process in the comparison of the visible working standards to the trap detectors is the relative experimental standard deviation of the weighted mean of multiple measurements using Eq. (1). This uncertainty is an order of magnitude smaller than the noise present in the source because of the common mode noise rejection obtained by using the monitor detector. The relative standard uncertainty in the absolute spectral power response of the trap detectors (from HACR) is 0.03 %. The electrical signals are measured with a current-to-voltage (transimpedance) amplifier and a digital voltmeter. The amplifier is calibrated and characterized for linearity, noise performance, and gain factor.

The monochromator wavelength calibration is determined using atomic emission lines from several commercial low pressure spectral lamps. The slit scattering function is the transmission function of the monochromator, and is typically measured by scanning the monochromator with a fixed wavelength laser source(s) over the entire spectral range of interest [25]. The (spectral) stray light is the amount of light outside the bandpass of the monochromator. We estimated this by multiplying the slit scattering function by the source spectral distribution and the spectral responsivity of the silicon photodiodes. At worst, with the monochromator tuned to ≈ 400 nm, the fraction of the signal due to stray light is 0.01 %.

The (spatial) scattered light is the light scattered outside the nominal beam diameter at the detector position. This was measured using a silicon photodiode with a small 0.6 mm aperture fixed on the front. The photodiode and aperture scanned a 12 mm by 12 mm area centered on, and in the focal plane of, the beam. This was done at several wavelengths in the 400 nm to 950 nm spectral range and the integrated power outside a diameter of 2 mm is no greater than 0.05 % of the total beam power.

The diffuse stray light (i.e., from the room and other sources) is the light scattered into the detectors that is not from the monochromator. This was measured by covering a detector with a light-tight cover and comparing that signal to the shutter closed signal. Although there was no detectable difference between the two signals under the conditions described, the diffuse stray light was assigned a relative standard uncertainty of not greater than 0.001 %. This signal, as well as the bias signal from the amplifiers, is normally subtracted from the shutter open signal, but in general it can contribute to the overall noise in the final result.

The combined effect of the beam nonuniformity and detector nonuniformity was measured by scanning a spot of diameter 1.1 mm across the each detector in the 400 nm to 950 nm spectral range. The uncertainty due to the beam and detector nonuniformities was estimated as the largest measured nonuniformity in the center 5 mm by 5 mm area of any working standard or trap detector.

The polarization sensitivity was estimated to be less than 0.01 %. The working standards responsivity non-linearity has been estimated to be 0.01 % over the power range from the comparator to the HACR. The temperature variations in the laboratory are on the order of 1 8C, which corresponds to a 0.02 % relative standard uncertainty in the response [26]. Long-term stability in spectral responsivity has been estimated to be 0.05 %. The uncertainties are combined following the method described in Ref. [24] (quadrature) to obtain a relative combined standard uncertainty of 0.11 % and a relative expanded uncertainty (*k* = 2) of 0.22 %.

## 6. Current Capabilities

**Fig. 3:**
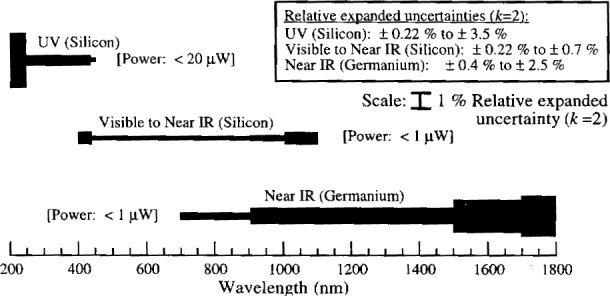
NIST UV, visible, and near IR absolute spectral responsivity measurement capabilities.

Even though this paper deals only with the scale from 406 nm to 920 nm, the present capabilities for photodetector absolute spectral power response measurements are from 200 nm to 1800 nm. The detector scale relative expanded uncertainties versus wavelength and working standards are shown in Fig. 3.

Future papers and NIST publications will describe the extension from the “visible” (406 nm to 920 nm) to the ultraviolet (200 nm) and near infrared (1800 nm) regions of the spectrum. NIST has recently added several new detector measurement test numbers. These are:

**Table:** 

Test #	Description
39071S	UV Silicon Photodiodes
39072S	Retest of UV Silicon Photodiodes
39073S	Visible/Near IR Silicon Photodiodes
39074S	Retest of Visible/Near IR Silicon Photodiodes
39075S	Special Tests of Near Infrared Photodiodes
39080S	Special Tests of Radiometric Detectors
39081S	Special Tests of Photodetector Spatial Responsivity Uniformity

### UV Silicon Photodiodes (39071S)

NIST will supply the customer with a silicon photodiode characterized in the ultraviolet (UV) spectral region. The UV silicon photodiode includes the measured spectral responsivity (in the unit A/W) from 200 nm to 450 nm in 5 nm steps and the relative changes in response over the photosensitive area at 350 nm. The photosensitive area of the photodiodes is underfilled for the measurements with a beam of diameter 1.5 mm. The spectral responsivity is measured at radiant power levels of less than 20 mW. The bandpass of the measurement is 4 nm. The relative expanded uncertainty (*k* = 2) ranges from 0.2 % to 3.5 %, depending on the wavelength.

### Retest of UV Silicon Photodiodes (39072S)

Special tests of previously supplied NIST ultraviolet (UV) silicon photodiodes are performed by measuring spectral responsivity (in the unit A/W) from 200 nm to 450 nm.

### Visible/Near IR Silicon Photodiodes (39073S)

NIST will supply the customer with a silicon photodiode characterized in the visible to near infrared (near IR) spectral region. The spectral response (in the unit A/W) of the visible to near infrared silicon photodiode is measured from 400 nm to 1100 nm in 5 nm steps. The relative change in response over the photosensitive area is also measured at 500 nm. The photosensitive area of the photodiodes is underfilled for the measurements with a beam of diameter 1.1 mm. The spectral responsivity is measured at radiant power levels of less than 1 μW. The bandpass of the measurement is 4 nm. The relative expanded uncertainty (*k* = 2) ranges from 0.2 % to 0.7 %, depending on the wavelength.

### Retest of Visible/Near IR Silicon Photodiodes (39074S)

Special tests of previously supplied NIST visible to near infrared silicon photodiodes are performed by measuring spectral responsivity (in the unit A/W) from 400 nm to 1100 nm.

### Special Tests of Near Infrared Photodiodes (39075S)

Special tests of customer supplied near infrared photodiodes are performed by measuring spectral responsivity (in the unit A/W) from 700 nm to 1800 nm. A beam of diameter 1.1 mm is centered on and under-fills the photosensitive area. The spectral response is measured at radiant power levels of less than 1 μW. The bandpass of the measurement is 4 nm. The relative expanded uncertainty (*k* = 2) ranges from 0.4 % to 2.5 %, depending on the wavelength. Customers should contact one of the authors to discuss details before submitting a formal request.

### Special Tests of Radiometric Detectors (39080S)

Special tests of radiometric detectors generally used in the ultraviolet, visible, and infrared regions of the spectrum can be performed. Responsivity of detectors can be measured between 200 nm and 1800 nm at power levels less than 1 μW (less than 20 μW between 200 nm and 450 nm). Examples of detector characteristics that can be determined in a special test include spectral responsivity (expressed in the unit A/W), quantum efficiency (electrons per photon), or linearity. The relative expanded uncertainty (*k* = 2) ranges from 0.2 % to 3.5 %, depending on the wavelength. Measurements of the relative change in response over the photosensitive area (spatial response uniformity) are conducted under Test Number 39081S. Since special tests of this type are unique, details of the tests should be discussed with one of the authors before submitting a formal request.

### Special Tests of Photodetector Spatial Responsivity Uniformity (39081S)

Special tests of customer supplied photodetectors can be performed by measuring the relative changes in responsivity across the photosensitive area (spatial responsivity uniformity). The uniformity is typically measured at a single wavelength in 0.5 mm increments with a beam diameter of 1.5 mm in the 200 nm to 400 nm spectral region, and a beam of diameter 1.1 mm in the 400 nm to 1800 nm spectral region. Customers should contact one of the authors to discuss details before submitting a formal request.

## 7. Quality System

The spectroradiometric detector measurements described here are part of the NIST Radiometric Physics Division calibration services that are currently being brought into compliance with ANSI/NCSL Z540-1-1994 [27, 28]. Although quality procedures were previously in place, they varied from calibration service to calibration service within the Division. The quality control procedures were typically limited to only the technical aspects of the measurements, for example, the yearly calibration of voltmeters, the use of multiple working standards, and their routine rotation.

The goal of the ANSI/NCSL Z540-1-1994 compliance project (which began in 1993) is to unify all the calibration services offered by the Radiometric Physics Division with standard formats and similar procedures. Balancing functionality and bureaucracy was a concern from the start. Efforts are directed toward developing a useful and practical quality system. Excessively sophisticated and complex procedures are avoided, along with redundant documentation. Tools such as checklists, forms, and flowcharts are used where applicable.

## 8. Future

At present the detector scale is extended with a room temperature pyroelectric detector. The pyroelectric detector has a low signal-to-noise ratio at the power levels available in the comparator. We have begun to develop a special cryogenic bolometer for this purpose, which will be usable down to a few pW [29, 30]. We are also developing new trap detectors suitable for use in the near IR and UV, which will be calibrated at selected laser wavelengths using the HACR.

## Figures and Tables

**Table 1:** Uncertainty budget for Vis/NIR spectral comparator facility absolute spectral responsivity measurements from 406 nm to 920 nm

Uncertainty origin	Relative standard uncertainty (%)	
	Type A	Type B
Relative experimental standard		
deviation of the weighted mean	0.05	
Trap calibration with HACR		0.03
Electrical current-to-voltage conversion	0.003	
Voltmeter calibration		0.005
Wavelength calibration		0.02
Stray light (spectral)		0.01
Scattered light (spatial)		0.05
Diffuse stray light		0.001
Beam and detector nonuniformity		0.05
Polarization sensitivity		0.01
Responsivity non-linearity		0.01
Temperature variations		0.02
Long-term stability		0.05
	
Relative combined standard uncertainty	0.11	
Relative expanded uncertainty (*k* = 2)	0.22	

## Footnotes

1 This designation of “visible” is rather arbitrary (though the 406 nm to 920 nm region does include most of the visible region) and made here for convenience and ease of reading and not for any technical reason.

2 The term “3 standard deviation estimate” or “3*σ* ” in the context of this paper is the estimated standard deviation multiplied by 3. Prior work at NIST was generally reported with “3*σ* ” uncertainties.

3 Certain commercial equipment, instruments, or materials are identified in this paper to foster understanding. Such identification does not imply recommendation or endorsement by the National Institute of Standards and Technology, nor does it imply that the materials or equipment identified are necessarily the best available for the purpose.
